# Mapping and Detection of Genes Related to Trichome Development in Black Gram (*Vigna mungo* (L.) Hepper)

**DOI:** 10.3390/genes15030308

**Published:** 2024-02-27

**Authors:** Dan Gong, Jianling Li, Suhua Wang, Aihua Sha, Lixia Wang

**Affiliations:** 1 Key Laboratory Grain Crop Genetic Resources Evaluation and Utilization, Ministry of Agriculture and Rural Affairs, Institute of Crop Sciences, Chinese Academy of Agricultural Sciences (ICS-CAAS), Beijing 100081, China; 2 College of Agriculture, Yangtze University, Jingzhou 434025, China

**Keywords:** black gram, trichome, gene mapping, candidate genes

## Abstract

Black gram (*Vigna mungo* (L.) Hepper) is a pulses crop with good digestible protein and a high carbohydrate content, so it is widely consumed as human food and animal feed. Trichomes are large, specialized epidermal cells that confer advantages on plants under biotic and abiotic stresses. Genes regulating the development of trichomes are well characterized in *Arabidopsis* and tomato. However, little is known about trichome development in black gram. In this study, a high-density map with 5734 bin markers using an F_2_ population derived from a trichome-bearing and a glabrous cultivar of black gram was constructed, and a major quantitative trait locus (QTL) related to trichomes was identified. Six candidate genes were located in the mapped interval region. Fourteen single-nucleotide polymorphisms (SNPs) or insertion/deletions (indels) were associated with those genes. One indel was located in the coding region of the gene designated as *Scaffold_9372_HRSCAF_11447.164*. Real-time quantitative PCR (qPCR) analysis demonstrated that only one candidate gene, *Scaffold_9372_HRSCAF_11447.166*, was differentially expressed in the stem between the two parental lines. These two candidate genes encoded the RNA polymerase-associated protein Rtf1 and Bromodomain adjacent to zinc finger domain protein 1A (BAZ1A). These results provide insights into the regulation of trichome development in black gram. The candidate genes may be useful for creating transgenic plants with improved stress resistance and for developing molecular markers for trichome selection in black gram breeding programs.

## 1. Introduction

Trichomes are the first line of defense for plants in that they protect stems and leaves against pathogen or herbivore attack [1,2,3]. They also play important roles in the induction of pollination, resistance in extreme environments, protection against ultraviolet radiation and high temperatures, prevention of mechanical damage, and tolerance to heavy metals, drought, and salinity [4]. Trichomes are large, specialized epidermal cells that are distributed on various organs of a plant’s above-ground parts. They are involved in development and help to prevent excess transpiration [4,5]. There are two types of trichomes: non-glandular epidermal trichomes (bristles and hairs) and glandular trichomes. Glandular trichomes are epidermal outgrowths in which large quantities of specialized metabolites are biosynthesized and stored. These metabolites function in the protection of plants against biotic and abiotic stresses [6]. Non-glandular trichomes are widely distributed on the leaf blade or concentrated along veins or in vein axils on the abaxial leaf surface. They can benefit mites by providing shelter from their natural enemies as well as by increasing the availability of alternative food sources such as pollen [7].

Genes regulating the development of non-glandular trichomes in *Arabidopsis thaliana* have been well characterized, and it is considered that the transcription factors of R2R3 MYB encoded by *GLABRA1 (GL1)* and *MYB23* initiate the trichome. Then, the bHLH transcription factors GLABRA3 (GL3) and ENHANCER OF GL3 (EGL3) interact with the WD-40 repeat-containing protein TRANSPARENT TESTA GLABRA1 (TTG1) to form the MBW (MYB-bHLH-WD40) complex, which activates the expression of the homeodomain–leucine zipper (HD ZIP) protein GLABRA2 (GL2) to promote trichome formation. The negative regulators *Caprice (CPC)*, *Tripty Chon (TRY)*, *Enhancer* of *TRY* and *CPC1 (ETC1)*, and *Enhancer of TRY and CPC2 (ETC2)* function redundantly to negatively regulate the development of trichome through the repression of *GL3*, *EGL3*, and *TTG1* [4,8]. The hormones gibberellin, cytokinin, jasmonic acid, and brassinolide promote the occurrence of trichomes, while salicylic acid (SA) inhibits the growth and development of trichomes by interfering with the jasmonate pathway [4]. Genes involved in the formation and development of glandular trichomes have been studied in detail in *Solanum* species, too. For instance, the *GL2* homolog *Woolly (Wo)* affects trichome development by regulating the expression of *SlCycB2* in tomato. The C2H2 zinc-finger protein Hair is involved in the initiation of type I glandular trichomes, and the *Hairless* gene encoding a subunit of the WAVE regulatory complex plays a role in the regulation of specialized metabolism in glandular trichomes [6]. In addition, a series of genes that regulate trichome traits in other crops were identified, such as *brphl1* in rapeseed (*Brassica rapa* L.) [9], *GoSTR* in cotton (*Gossypium herbaceum* L.) [10], and *Csa6M514870 s* in cucumber (*Cucumis sativus* L.) [11].

There are QTLs related to trichome that have been reported in different species. One QTL was demonstrated to control both predatory mite abundance and leaf trichomes in *Vitis* [7], while three QTLs related to stem trichome were observed in cotton [12]. In soybean (*Glycine max* L.), a total of 10 and 9 QTLs for trichome length and density were identified, respectively, and three and four candidate genes were estimated, respectively [13]. 

Black gram (*V. mungo* (L.) *Hepper*) is a pulses crop belonging to the genus *Vigna Savi* (subgenus *Ceratotropis*), together with mungbean (*Vigna radiata* (L.) R. Wilczek), rice bean (*Vigna umbellata* (Thunb.) Ohwi & Ohashi) and adzuki bean (*Vigna angularis* (Willd.) Ohwi & Ohashi). High ratios of synteny on the genomic level among these species have been observed [14]. Black gram is widely cultivated in Asian countries, especially India, Bangladesh, Sri Lanka, Myanmar, and Thailand. It is an excellent source of human food and animal feed because its seeds contain a high ratio of good-quality protein and carbohydrates, a low level of fat (1.5%), and certain minerals, amino acids, and vitamins. Black gram also plays a significant role in soil fertility by fixing atmospheric nitrogen and usually used as an intercrop with gramineous crops. It is suitable for dry land farming and is predominantly grown as an intercrop or as a sole crop under residual moisture conditions after rice harvest because it is tolerant to drought [15,16]. Although black gram is severely affected by various biotic and abiotic stresses, it has a fairly higher ability of resistance to bruchid than any other *Vigna* crops, which is beneficial for breeding in mungbean and adzuki bean by inter-specific crossing or DNA molecular technology.

As a self-pollinating diploid species (2n = 2x = 22), the genome size of black gram was estimated to be 574 Mb. Several chromosome-scale assemblies of genome for this species have been reported [9,10], and these have accelerated the studies of comparative genomics and phylogenetics in legume species and could lay an important base for identifying and explicating target genes. Trichome is the classical trait for most black gram accessions. It is said that trichomes are usually advantageous in terms of defense against pathogens and herbivores, but little is known about the QTLs or genes controlling the development of trichome in black gram. Therefore, in the present study, we conducted a high-density map based on an F_2_ population derived from a trichome-bearing and a glabrous cultivar, and a major QTL related to trichomes was identified based on the evaluation of the phenotypes of each F_2_ line. Further analyses showed that genes encoding RNA-polymerase-associated protein Rtf1 and Bromodomain adjacent to zinc-finger domain protein 1A (BAZ1A) might be the candidate genes. These candidate genes may be useful for creating transgenic plants as well as for developing molecular markers to select for trichomes in black gram, with an overall aim to generate new lines with increased tolerance to biotic or abiotic stresses.

## 2. Materials and Methods

### 2.1. Mapping Population Construction and Sampling

A cross between the trichome-bearing cultivar (P1) as the male parent and the glabrous cultivar (P2) as the female parent was made in 2019. F_1_ lines were planted in 2020, and an F_2_ population with 193 lines was developed in Beijing in the summer of 2021. Fresh and young leaves were sampled from the 193 F_2_ lines and the parents. Each leaf sample was frozen in liquid nitrogen and then stored at −80 °C until DNA extraction.

### 2.2. Phenotype Analysis

In 2021, the presence or absence of trichomes on the 193 F_2_ plants and their parents in the field in Beijing was determined by visual observation. The phenotypes of F_2_ individuals were confirmed in 2022 by evaluating 30 F_3_ plants derived from each F_2_ plant.

### 2.3. Genomic Resequencing by Illumina

Genomic DNA was prepared using the CTAB method and was quantified using agarose gel electrophoresis and a Qubit flex instrument (Thermo Fisher, Waltham, MA, USA). The total genomic DNA was first broken into ~300 bp fragments for paired-end library construction according to the standard protocol provided by Illumina (San Diego, CA, USA). After quality detection, the libraries were further used for paired-end sequencing with 150 bp read length through the HiSeq X platform (Illumina).

### 2.4. Single-Nucleotide Polymorphism Calling and Bin Map Construction

Firstly, the residual adapter and low-quality sequences were removed from the raw data in fasta format using the fastp program (https://github.com/OpenGene/fastp, accessed on 1 January 2024). Then, the high-quality reads were aligned to the reference genome of black gram (Vigna mungo var. mungo-NCBI-NLM [15] using BWA version 0.7.5a with default parameters [17]. Variants from all samples were obtained using GATKProgram [18], and a Perl script was applied to detect single-nucleotide polymorphisms (SNPs) [19]. Alleles with extreme distortion (chi-square test, *p*-value < 1 × 10^−8^) were discarded. High-quality variation loci met the following criteria: (1) parental alleles were homozygous and different; and (2) the minor allele frequency in the F_2_ population was greater than 0.05, and the missing rate was less than 0.1.

The bin map was constructed using the sliding window method as described by Huang et al. [20] with minor modifications. Each 15 SNP window size was scanned with a sliding step of 1 SNP. For each line in each window, the genotype was defined as follows: (1) homologous P1 genotype: SNPP1:SNPP2 ≥ 13; (2) homologous P2 genotype: SNPP2:SNPP1 ≥ 13; (3) heterozygous genotype: SNPP1: SNPP2 or SNPP2: SNPP1 less than 13. The determination of breakpoints and the construction of the bin map were conducted according to the methods of Huang et al. [20].

### 2.5. QTL Mapping and Candidate Gene Identification

QTL were identified using the inclusive composite interval mapping method [21]. A significant LOD threshold for mapping was determined with a 3000 permutations test (*p* = 0.01). The genes located in a 3 LOD-drop interval were predicted as candidate genes.

### 2.6. Validation of Candidate Genes by Real-Time Quantitative PCR

Total RNAs were isolated from the stems and pods of the two parental lines with an RNA extraction kit (Huayueyang, Beijing, China), and then, 5 µg total RNA was used to synthesize first-strand cDNA with the Super Script First-Strand cDNA Synthesis Kit (Invitrogen, Carlsbad, CA, USA). The real-time quantitative PCR (qPCR) analyses were conducted using the Bio-Rad CFX Connnect™ Optics Module System (Bio-Rad, Hercules, CA, USA). Each 10 μL reaction mixture consisted of 5 μL SYBR Premix Ex Taq (Tiangen, Beijing, China), 0.5 μL of each primer (10 μM), 2 μL cDNA template, and 2 μL RNase-free water. Three biological replicates and two technical replicates were analyzed for each gene. The primers for each gene are listed in Table S1. The reaction program was set as follows: 95 °C for 30 s, followed by 40 cycles of 95 °C for 5 s and 60 °C for 30 s. Melting curve analyses were conducted to confirm the specificity of primers. The 2^−ΔΔCt^ method was used to calculate the relative transcript levels of each gene [22]. *Actin* (accession JZ078743) was used as the internal reference gene, as described by Kundu et al. [23].

## 3. Results

### 3.1. Phenotypic Variations among the Parents and the F_2_ Population

**Figure 1:**
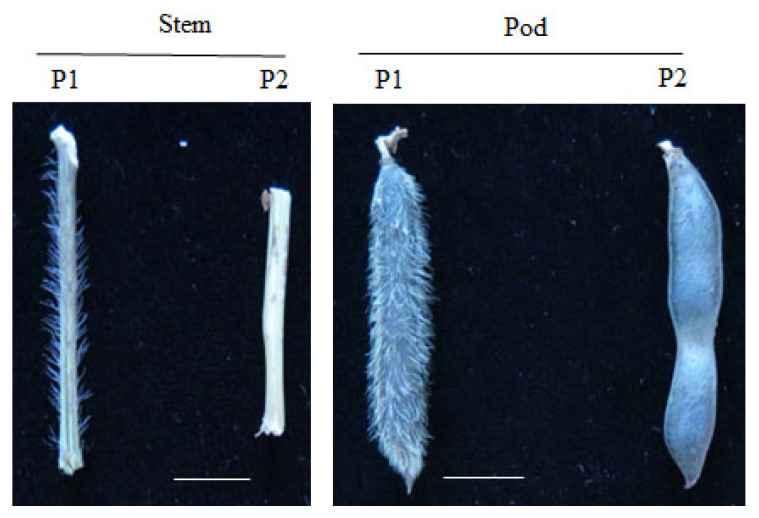
Trichome-bearing and glabrous phenotypes of two parents of black gram. Scale = 1 cm.

The P1 parent had trichomes on the stems and pods, whereas the P2 parent had glabrous stems and pods (Figure 1). Observations of the F_2_ population indicated that 146 out of 193 lines bore trichomes, whereas 47 out of 193 lines were glabrous (Table S2). The results of the chi-square test (χ^2^ = 0.028 < χ^2^ 0.05(1) = 3.84 (*p* > 0.05)) implied that one major positive gene controls the presence of trichomes in black gram. Analyses based on the phenotypes of F_3_ lines confirmed this result.

### 3.2. Sequencing, SNP Identification, and Bin Map Construction

**Figure 2:**
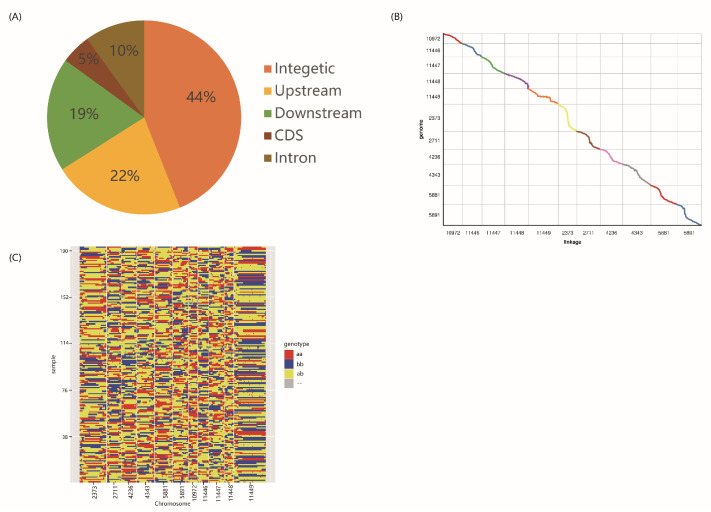
The distribution of SNP and the recombination bin map of F_2_ population of black gram. (**A**) Distribution of SNPs/indels among genomic regions. (**B**) Genetic linkage map of 5734 bins inferred from 178,168 high-quality variations (SNPs/indels). (**C**) Collinearity between linkage groups and chromosomes.

A whole-genome resequencing strategy was applied to construct paired-end libraries for both of the parents and their 193 F_2_ progeny. Approximately 446 Gb of clean data (Q30 > 90%) were generated, resulting from 1519 million reads. In total, more than 33 million reads were obtained for each of the two parents, whereas the number of reads obtained for the F_2_ lines ranged from 0.64 to 0.95 million (Table S3). The coverage rate, mapped reads rate, sequencing depth, and other results indicative of alignment to the reference genome are shown in Table S3. In particular, the coverage rate associated with P1 and P2 was 98.0% and 99.4%, respectively, whereas it ranged from 82.4% to 95.21% with an average of 90.27% in the F_2_ population (Table S3). The coverage rate in F_2_ lines was relatively low, probably because of the poor quality of the reference genome or because of the distant relationship between the F_2_ lines and the cultivar used for assembly of the reference genome. The sequencing depth was 14×, 13×, and an average of 2.92× for P1, P2, and the F_2_ lines, respectively. Using Samtools and GATK, more than 1 million variations (SNPs and indels) were identified from all the samples. After excluding non-effective variation loci, a total of 576,100 SNPs were retained, of which 44% were located in intergenic regions and only ~5% in gene-coding regions (Figure 2A).

### 3.3. Construction of Physical Recombination Maps and High-Density Genetic Linkage Map

To avoid errors related to the low genome-coverage rates of the F_2_ population’s sequences, a sliding window with 15 consecutive SNPs was used to identify recombined breakpoints more accurately. The physical map of 193 F_2_ lines was constructed based on the recombination in each progeny (Figure 2C). After that, all chromosomes of the 193 F_2_ lines were aligned and compared at a minimum of 100 kb intervals. Finally, 5734 bin markers inferred from 178,168 high-quality SNPs in the 193 lines were obtained and distributed on eleven linkage groups (LG) (Figure 2C, Table 1 and Table S4).

**Figure 3:**
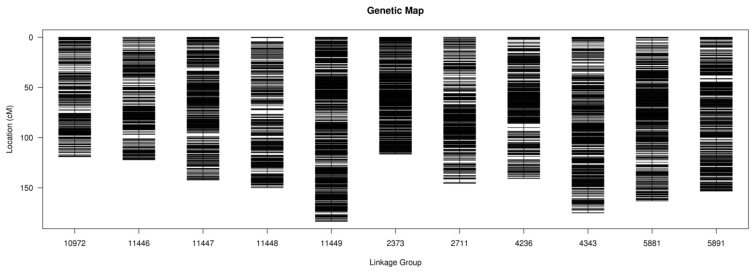
Genetic linkage map of black gram obtained from an F_2_ population.

The total length of the 11 LGs was 1611.43 cM, and the marker interval across linkage groups ranged from 0.14 to 0.50 cM with an average value at 0.28 cM (Table 1). LG11448 had the smallest number of markers (301), and LG2373 had the shortest genetic length (116.59 cM), whereas LG11449 had the highest number of markers (981) and the longest genetic length (183.49 cM) (Table 1 and Figure 3). There were no gaps larger than 5 cM on the linkage group, and the largest gap was 4.36 cM, located on LG11448 (Table 1). In a collinearity analysis, Spearman’s coefficient between linkage groups and chromosomes was close to 1 (Figure 2B, Table S5).

### 3.4. QTL Mapping and Prediction of Candidate Genes

**Figure 4:**
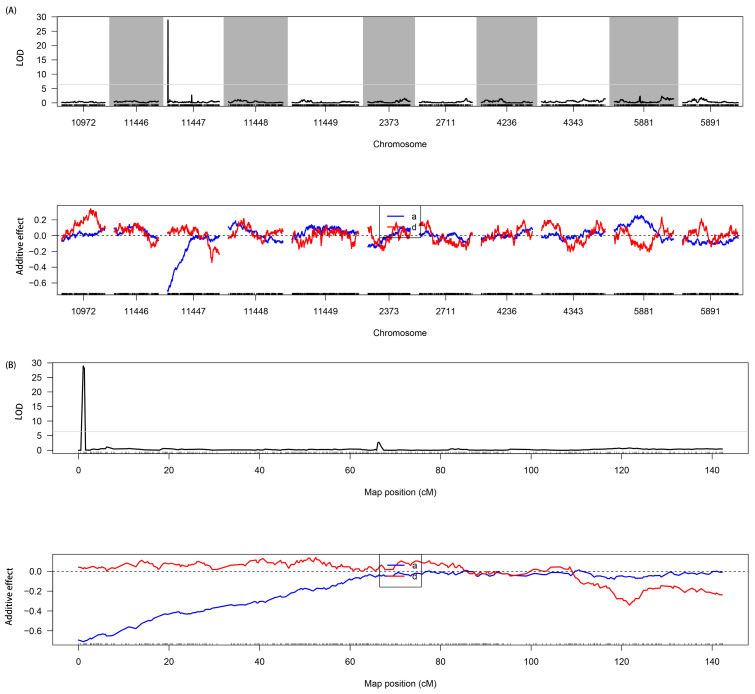
QTL mapping to detect target regions related to trichomes in black gram. (**A**) Genome mapping for QTL. (**B**) QTL location on LG11447. a, additive effect; d, dominant effect.

Genetic mapping showed that one major QTL related to trichome was identified on LG11447 (Figure 4A,B). This QTL explained 50.89% of phenotypic variation and had a LOD value of 28.89.

To identify the candidate gene(s) related to trichomes, coding sequences in the genomic region associated with the QTL were examined to determine their predicted function, according to the *V. mungo* cultivated variety CN 80 reference genome annotation database [15]. The QTL spanned a physical interval of ~47 kb and contained six genes, which encoded stAR-related lipid transfer protein 7, RNA polymerase-associated protein Rtf1, CDI (cadmium ^2+^ induced), Bromodomain adjacent to zinc-finger domain protein 1A, histone demethylase, and RGF1 INDUCIBLE TRANSCRIPTION FACTOR 1 (Table 2).

To validate the candidate genes, the mutation type of the SNPs or indels associated with each candidate gene were further analyzed (Table 3). As a result, there were 14 polymorphic SNPs or indels between the two parents located in five genes but not in *Scaffold_9372_HRSCAF_11447.167* (simply referred to *SCA-167*, with other genes named in the same way). Most of the SNPs or indels were located in non-coding DNA regions, whereas one indel was located in the coding region of *SCA-164*.

### 3.5. qPCR Validation

**Figure 5:**
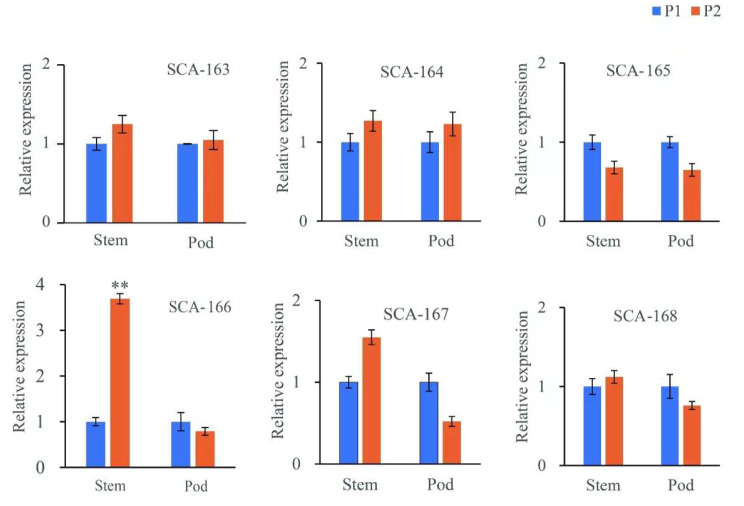
Transcript levels of six candidate genes related with trichome in stems and pods of two parents of black gram, as detected by qPCR. Actin was used as the internal reference gene. *SCA-163* to *SCA-168* were *Scaffold_9372_HRSCAF_11447.163*, *Scaffold_9372_HRSCAF_11447.164*, *Scaffold_9372_HRSCAF_11447.165*, *Scaffold_9372_HRSCAF_11447.166*, *Scaffold_9372_HRSCAF_11447.167*, and *Scaffold_9372_HRSCAF_11447.168*, respectively. ** means the significance difference at *p* < 0.001.

Because the P1 parent only had trichomes on the stems and pods, the transcript levels of the six candidate genes in the stems and pods of P1 and P2 parents were detected by qPCR. Of the six candidate genes, only *SCA-166* was down-regulated significantly in the stem of the P1 parent compared with the P2 parent. No significant differences were observed in the transcript levels of the other candidate genes in the stem and pod between P1 and P2 (Figure 5).

Finally, *SCA-164* and *SCA-166* were identified as the two candidate genes related to trichomes in black gram: *SCA-164* because it had one SNP located in its coding region, whereas all the other SNPs or indels associated with candidate genes were located in introns or upstream or downstream of the coding region, and *SCA-166* because it was differentially expressed between the two parents.

## 4. Discussion

Trichomes offer natural protection against pests and diseases in plants. As a minor species in the *Vigna* genus, black gram has the highest density of trichomes among all the *Vigna* crops, and this may confer a high level of protection against many pests and diseases, especially bruchids. Mining for genes related to trichomes and determining the mechanism of trichome development in black gram might be useful for generating new, stress-tolerant lines by inter-specific crossing or genetic modification, especially for related crops such mungbean and adzuki bean, which are most susceptible to bruchids during storage. Here, a high-density map with 5734 bin markers was constructed based on an F_2_ population. Only one major QTL related to trichomes was identified with a 28.89 LOD value, which explained 50.89% of phenotypic variation, indicating that the trichome trait has a great inheritance in black gram, which is agreeable with the segregation of the presence of trichomes in the progeny. It was demonstrated that the stem trichome development was simpler than leaf trichome in genetics, and only three QTLs for stem trichome were identified in cotton [10]. That is why only one major QTL was identified in our study due to the trichomes mainly being in the stem and pod of black gram. Fourteen SNPS or indels associated with the six candidate genes located in the QTL region were detected. However, only one indel was located in a gene-coding region. The indel in the coding sequence of *SCA-164* resulted in a frame-shift mutant. The SNPs or indels associated with other candidate genes were located in non-coding regions. Next, the transcript levels of all six genes in the QTL region were detected in the two parents by qPCR. The results demonstrated that only *SCA-166* was down-regulated in the stem of the trichome-bearing parent P1 compared with that of the glabrous parent P2. This result suggests that the down-regulation of *SCA-166* promotes trichome development. Therefore, *SCA-164* and *SCA-166* were identified as potential candidate genes related to trichomes in black gram. It was suggested that the glabrous stems were due to the insertion of TE in HD1 on Chr. 06, whereas the glabrous stems were due to gaining-function of the gene on Chr. 24 in cotton [10]. *GoSTR*, a homolog of GL2-interacting repressors *AtGIR1* and *AtGIR2*, encodes a hypothetical protein with only 84 amino acids, which inhibits the long hairs to form in cotton stem [12]. Overexpression of *SlCycB2*, the tomato homolog of *AtGIR2*, was found to cause the glabrous stems of tomato [24]. *SCA-164* encodes the RNA polymerase-associated protein Rtf1. Rft1 is a multifunctional elongation factor that plays roles in promoting co-transcriptional histone modifications, RNAPII (RNA polymerase II) elongation, and mRNA processing [25,26]. *SCA-166* encodes BAZ1A. The BAZ1 protein is involved in chromatin remodeling and is recruited to participate in global genome nucleotide excision repair after DNA damage caused by ultraviolet light in conjunction with several other proteins, including the histone acetylase HBO1 [27]. The fact that both *SCA-164* and *SCA-166* encode proteins involved in histone modification suggests that this process may be an important part of the regulation of trichome development in black gram. Indeed, histone modification has been reported to be involved in the regulation of trichome development in other plants. Chromatin assembly factor CAF-1 is a histone chaperone facilitating chromatin formation and the maintenance of specific chromatin states. It has been reported that mutations of *CAF-1* result in the formation of trichomes with supernumerary branches [28]. HAIRPLUS (HAP) is involved in histone tail modifications and has been shown to control the density of glandular trichome density in tomato [29]. In *Arabidopsis*, a mutation of *SET Domain Group 26* (*SDG26*), encoding a histone lysine methyltransferase, increased the number of trichomes and the trichome density on rosette leaves [4]. Mutations of the histone acetyltransferase gene *GCN5* and its associated transcriptional coactivator *ADA2b* were found to alter the number and patterning of trichome branches, resulting in elongation of the trichome stalk [30]. Further research including experimental analyses is required to determine whether and how *SCA-164* and *SCA-166* regulate trichome development in black gram.

## 5. Conclusions

In this study, one major QTL controlling trichomes in black gram was detected from a high-density genetic linkage map with 5734 bin markers, and two potential candidate genes involved in histone modification were identified. These results might be helpful for investigating the regulatory mechanism of trichome development in black gram. The candidate genes have potential applications in generating transgenic plants with improved stress resistance as well as in the development of molecular markers to select for trichomes in black gram breeding programs.

## Figures and Tables

**Table 1:** Summary information for 11 linkage groups (LGs) of black gram detected in this study.

Linkages	Total Bin Marker	SNP Numbers	Total Distance (cM)	Average Distance (cM)	Max Gap (cM)	Gaps < 5cM (%)
LG2373	835	34,278	116.59	0.14	1.3	100%
LG2711	446	14,688	145.62	0.33	3.22	100%
LG4236	480	17,664	140.77	0.29	4.05	100%
LG4343	545	16,824	175.06	0.32	2.65	100%
LG5881	559	19,318	163.13	0.29	2.39	100%
LG5891	479	15,862	153.28	0.32	3.22	100%
LG10972	293	10,189	119.09	0.41	2.65	100%
LG11446	345	10,877	122.16	0.36	4.31	100%
LG11447	470	15,522	142.25	0.3	3.53	100%
LG11448	301	7296	150	0.5	4.36	100%
LG11449	981	21,940	183.49	0.19	2.13	100%

**Table 2:** Candidate genes related to trichome development within the QTL in black gram.

Gene	Gene Location	Annotation
Scaffold_9372_HRSCAF_11447.163	Chr6: 834,818–839,129	stAR-related lipid transfer protein 7
Scaffold_9372_HRSCAF_11447.164	Chr6: 842,193–847,000	RNA polymerase-associated protein Rtf1
Scaffold_9372_HRSCAF_11447.165	Chr6: 848,491–849,285	CDI (cadmium 2+ induced)
Scaffold_9372_HRSCAF_11447.166	Chr6: 858,351–865,191	Bromodomain adjacent to zinc-finger domain protein 1A
Scaffold_9372_HRSCAF_11447.167	Chr6: 867,211–869,064	Histone demethylase JARID1
Scaffold_9372_HRSCAF_11447.168	Chr6: 870,460–872,091	RGF1 INDUCIBLE TRANSCRIPTION FACTOR 1

**Table 3:** Mutation type of SNPs or indels associated with candidate genes in black gram.

Gene_Id	Position	P1 (Trichome)	P2 (Glabrous)	Location/Effect
Scaffold_9372_HRSCAF_11447.163	836,049	G	GT	Intron
	838,193	A	AT	Intron
	839,519	C	CAAAAGAAAAG	Downstream
	839,654	AT	A	Downstream
Scaffold_9372_HRSCAF_11447.164	842,357	TCCG	T	Codon_insertion
	843,363	AAAG	A	Intron
Scaffold_9372_HRSCAF_11447.165	848,154	A	ACAGAG	Upstream
	848,319	T	TA	Upstream
	849,984	AATT	A	Downstream
Scaffold_9372_HRSCAF_11447.166	855,253	T	TA	Upstream
	855,365	A	ATTTT	Upstream
Scaffold_9372_HRSCAF_11447.168	873,299	G	GT	Downstream
	873,465	T	TA	Downstream
	873,655	T	TATTATC	Downstream

## Data Availability

The data presented in this study are available on request from the corresponding author.

## Supplementary Materials

The following supporting information can be downloaded at: https://www.mdpi.com/article/10.3390/genes15030308/s1, Table S1: Primers used in the study; Table S2: Phenotype of parents and F2 lines; Table S3: The summary information of the sequencing and alignment results; Table S4: The spearman of collinearity analysis between linkage groups and chromosomes; Table S5: The spearman of collinearity analysis between linkage groups and chromosomes.
