# Supplementary material for: Mapping and Detection of Genes Related to Trichome Development in Black Gram (Vigna mungo (L.) Hepper)
Source: Genes (Basel). 2024 Feb 27;15(3):308. doi: 10.3390/genes15030308 (PMC10970695; doi:10.3390/genes15030308)
Supplement: Supplementary file 1 [file genes-15-00308-s001.zip › Table S1.pdf]

Table S1. Primers used in the study

Primer	Sequence (5' to 3')	Target	Product size (bp)
SCA-163-F	GGACGATGACTTCCGTCCTA	Scaffold_9372_HRSCAF_11447.163	229
SCA-163-R	TCTGGGCTTATCCCGTATTG		
SCA-164-F	CTGTCTCGCACTGAAATCCTGA	Scaffold_9372_HRSCAF_11447.164	207
SCA-164-R	ACTCCCCACTTGCACTGAGA		
SCA-165-F	AAGATGGATGGGGCAGTGC	Scaffold_9372_HRSCAF_11447.165	274
SCA-165-R	CAAGCTTCAAACCATGGCCC		
SCA-166-F	GGAGGACTTGGGAACGATGG	Scaffold_9372_HRSCAF_11447.166	232
SCA-166-R	CCTGTCTACTGGCCCGACTA		
SCA-167-F	TGGCTTTCTTCTGGGTGGTG	Scaffold_9372_HRSCAF_11447.167	223
SCA-167-R	ATTGCCACCTCTCAAACGGG		
SCA-168-F	CAACTGGGGTGATGAGAGGT	Scaffold_9372_HRSCAF_11447.168	289
SCA-168-R	AGCAAAGGGCTCCATTGTTA		
Actin-F	GTTCTGTTCCAGCCATCCAT	Actin	220
Actin-R	GTGGTGCGACAACCTTGATT		
