# Supplementary material for: Mapping and Detection of Genes Related to Trichome Development in Black Gram (Vigna mungo (L.) Hepper)
Source: Genes (Basel). 2024 Feb 27;15(3):308. doi: 10.3390/genes15030308 (PMC10970695; doi:10.3390/genes15030308)
Supplement: Supplementary file 1 [file genes-15-00308-s001.zip › Table S2.pdf]

Table S2. phenotype of parents and F_2 lines

Lines	Phenotype	Lines	Phenotype	Lines	Phenotype	Lines	Phenotype	Lines	Phenotype	Lines	Phenotype	Lines	Phenotype
P1	T	R27-1	T	R57-1	G	R87-1	T	R117-1	T	R146-1	G	R175-1	T
P2	G	R28-1	G	R58-1	T	R88-1	T	R118-1	T	R147-1	T	R176-1	T
R1-1	G	R29-1	T	R59-1	T	R89-1	T	R119-1	T	R148-1	G	R177-1	G
R2-1	T	R30-1	T	R60-1	G	R90-1	T	R120-1	T	R149-1	T	R178-1	G
R3-1	G	R31-1	T	R61-1	T	R91-1	G	R121-1	T	R150-1	T	R179-1	G
R4-1	T	R32-1	G	R62-1	T	R92-1	T	R122-1	T	R151-1	T	R180-1	G
R5-1	T	R33-1	T	R63-1	T	R93-1	T	R123-1	T	R152-1	T	R181-1	T
R6-1	T	R34-1	T	R64-1	T	R94-1	T	R124-1	T	R153-1	T	R182-1	T
R7-1	G	R35-1	T	R65-1	G	R95-1	T	R125-1	T	R154-1	T	R183-1	T
R8-1	G	R36-1	G	R66-1	G	R96-1	T	R126-1	G	R155-1	G	R184-1	T
R9-1	T	R38-1	G	R67-1	T	R97-1	T	R127-1	T	R156-1	T	R185-1	G
R10-1	T	R39-1	G	R68-1	G	R98-1	T	R128-1	T	R157-1	T	R186-1	G
R11-1	T	R40-1	T	R69-1	T	R99-1	T	R129-1	T	R158-1	T	R187-1	T
R12-1	G	R41-1	G	R70-1	T	R100-1	T	R131-1	T	R160-1	T	R188-1	T
R13-1	T	R43-1	G	R71-1	G	R101-1	T	R132-1	T	R161-1	T	R189-1	T
R14-1	T	R44-1	T	R72-1	T	R102-1	G	R133-1	T	R162-1	T	R190-1	T
R15-1	T	R45-1	G	R73-1	T	R103-1	T	R134-1	T	R163-1	T	R191-1	G
R16-1	T	R46-1	T	R74-1	T	R105-1	G	R135-1	G	R164-1	T	R192-1	T
R17-1	G	R47-1	T	R75-1	T	R106-1	G	R136-1	T	R165-1	T	R193-1	G
R18-1	T	R48-1	T	R76-1	T	R107-1	T	R137-1	T	R166-1	T	R194-1	T
R19-1	T	R49-1	T	R77-1	T	R108-1	T	R138-1	T	R167-1	G	R195-1	T
R20-1	T	R50-1	G	R78-1	T	R109-1	T	R139-1	T	R168-1	T	R196-1	T
R21-1	T	R51-1	T	R79-1	T	R110-1	T	R140-1	G	R169-1	T	R197-1	G
R22-1	T	R52-1	T	R80-1	T	R111-1	T	R141-1	T	R170-1	T	R198-1	T
R23-1	G	R53-1	T	R81-1	T	R112-1	T	R142-1	T	R171-1	G	R200-1	T
R24-1	T	R54-1	T	R83-1	T	R113-1	T	R143-1	T	R172-1	T	R201-1	T
R25-1	T	R55-1	T	R84-1	T	R114-1	T	R144-1	T	R173-1	G	R202-1	T
R26-1	T	R56-1	T	R86-1	T	R115-1	G	R145-1	G	R174-1	G		

T, Trichome; G, glabrous
