# Supplementary material for: Mapping and Detection of Genes Related to Trichome Development in Black Gram (Vigna mungo (L.) Hepper)
Source: Genes (Basel). 2024 Feb 27;15(3):308. doi: 10.3390/genes15030308 (PMC10970695; doi:10.3390/genes15030308)
Supplement: Supplementary file 1 [file genes-15-00308-s001.zip › Table S5.pdf]

Table S4. The spearman of collinearity analysis between linkage groups and chromosomes

LG ID Spearman	Spearman
LGScaffold_HRSCAF_5891	0.9999
LGScaffold_HRSCAF_2711	0.9978
LGScaffold_HRSCAF_2373	0.9991
LGScaffold_HRSCAF_11446	0.9996
LGScaffold_HRSCAF_11447	0.9999
LGScaffold_HRSCAF_4343	0.9995
LGScaffold_HRSCAF_4236	0.9982
LGScaffold_HRSCAF_10972	0.9997
LGScaffold_HRSCAF_5881	0.9998
LGScaffold_HRSCAF_11449	0.9924
LGScaffold_HRSCAF_11448	0.9991
